# New Horizon in the Treatment of Calcific Aortic Stenosis: How Far Can We Go?

**DOI:** 10.1016/j.jacasi.2024.08.017

**Published:** 2024-10-08

**Authors:** Qifeng Zhu, Stella Ng, Xianbao Liu, Jian’an Wang

**Affiliations:** a Department of Cardiology of The Second Affiliated Hospital, School of Medicine, Zhejiang University, Hangzhou, China; b State Key Laboratory of Transvascular Implantation Devices, Hangzhou, China; c Research Center for Life Science and Human Health, Binjiang Institute of Zhejiang University, Hangzhou, China; d Cardiovascular Key Laboratory of Zhejiang Province, Hangzhou, China

**Keywords:** aortic stenosis, calcium, treatment

Currently, the focus of interventional cardiology centers on the treatment of aortic stenosis (AS). Transcatheter aortic valve replacement (TAVR) and surgical aortic valve replacement (SAVR) have emerged as established therapeutic modalities for addressing symptomatic and severe AS. Notably, TAVR is a minimally invasive procedure that is associated with smaller incisions, faster recovery, and shorter hospital stays. Consequently, TAVR has undoubtedly become the mainstream therapy for calcific AS in high income countries, such as the United States, the United Kingdom, and Japan, surpassing SAVR in frequency in recent years. In developing countries, such as China, SAVR remains a more common option for patients with calcific AS, although the number of TAVR cases is rapidly increasing.

Nevertheless, significant subsets of patients remain ineligible for both TAVR and SAVR procedures: factors such as porcelain aorta, extensive calcification of the aortic annulus and left ventricular outflow tract, heightened risk of coronary obstruction (caused by sinus sequestration, bulky calcium on leaflet, and so on), and hypersensitivity to stent frame and other implanted materials are the main reasons to withhold for further intervention. Moreover, younger patients with less concomitant conditions (eg, women of child-bearing age) might also deny open surgery and possible multi-intervention because of personal and family considerations.

Calcific degeneration is the predominant etiology of AS in high income countries, and recent studies have demonstrated its prevalence in certain regions of Asia, such as China.^1^^,^^2^ Preliminary in vivo and in vitro studies have suggested that disruption of calcification on the leaflets could potentially reduce stenosis of the valve orifice.^3^ The technique primarily relies on the cavitation effect, which generates a shockwave with fracturing impacts on calcified tissue. Consequently, the concept of modifying aortic valve calcification through the cavitation effect holds promise for patients considered to be high risk for both TAVR and SAVR.

Despite the early attempt of ultrasonic aortic valve decalcification in the early 1990s, subsequent complications (eg, aortic regurgitation) and its invasive nature have significantly restricted its further expansion. Recently, Messas et al^4^ reported promising outcomes of a noninvasive ultrasound therapy (NIUT) for the treatment of AS. In this multinational, prospective cohort study, a total of 40 patients with symptomatic severe AS who were not suitable candidates for SAVR or TAVR underwent NIUT therapy. They directed focused ultrasounds toward a specified target within the aortic valve tissue, guided by real-time echocardiographic imaging. At the focal point, the formation and collapse of small bubbles induced a subsequent cavitation effect, leading to calcium modification. The mean procedural duration was 56 minutes, with a total mean acoustic power delivery of 5,540 W and a mean focal acoustic energy of 375 J/mm^2^. The procedure has demonstrated safety through a 30-day follow-up, and only a few patients experienced discomfort during the treatment. Three patients received retreatment within 6 months. Neurological function remained preserved among all patients during the follow-up period. In comparison to the baseline echocardiogram, aortic stenosis was mildly relieved (the AVA increased by 10% and mean pressure gradient decreased by 7%, both statistically significant), accompanied by the improvement in NYHA functional class. This noninvasive approach not only offers an alternative for treating symptomatic severe AS, but also presents an intriguing avenue for managing moderate AS and addressing the underexpansion of TAVR prostheses.

However, like any medical intervention, NIUT presents potential drawbacks. Adverse effects on adjacent structure such as the conduction system, coronary artery, and mitral valve need further elucidation in subsequent trials with larger sample sizes. Additionally, the treatment efficacy of NIUT appears to be notably reduced in human trials compared with animal and pulsatile flow test. This reduction can be attributed to the attenuation caused by the soft tissues and bones of the human body, emphasizing the need for long-term efficacy assessments in future follow-up studies.

Physicians engaged in the field of valvular heart disease have been actively exploring therapeutic approaches that do not involve the need for implantation or replacement. Hence, other approaches like transcatheter aortic valve lithotripsy, histotripsy, valve scoring, and leaflet splitting/laceration are currently under investigation.

Lithotripsy-assisted aortic valvuloplasty is conducted with a specialized balloon, whereby a radial shockwave is released from the center of the balloon, causing fragmentation of aortic valve calcification and improvement of leaflet immobility.^3^^,^^5^ We tested this approach in vitro and demonstrated that it could rupture calcium deposits and increase tissue compliance, which is expected to improve aortic valve hemodynamics. The subsequent first-in-man trial indicated its safety and efficacy among human calcific human aortic valve. Procedural safety and neurological function were well documented during follow-up, which was up to 6 months for all 7 patients who received lithotripsy-assisted aortic valvuloplasty in our center. Histotripsy shares a similar mechanism of lithotripsy, except that the ultrasonic wave was generated at the first place and subsequent shockwave energy and cavitation effect was delivered for histotripsy.^6^

With regard to the valve scoring device, it performs scoring on the aortic valve leaflet with the intention of creating multiple scoring lines, which has reported remarkable improvement of aortic valve orifice and hemodynamic parameters.^7^

As for leaflet splitting technology, the CATHEDRAL (CATHeter Electrosurgical Debulking and RemovAL) procedure involves the action of grasping the leaflet using a guidewire, followed by enclosing and detaching the leaflet or bulky calcification by electrosurgical force.^8^ Others consist of a splitting element and a positioning part, which penetrates the leaflet in a controlled manner and performs leaflet splitting by mechanical or electrosurgical force.^9^^,^^10^ It is to be noted that all of these approaches have been developed to address the need for coronary protection during the TAVR (particularly valve-in-valve) procedure, thus they would possibly lead to immediate aortic regurgitation by its inborn nature, which makes them more of a bridge therapy rather than a destination therapy.

Noninterventional therapeutics, such as drug targets (including low-density lipoprotein cholesterol, lipoprotein(a), mineral-binding matrix gla protein, and phosphate/calcium-metabolism associated targets), have been identified, and ongoing clinical trials might further clarify their potential therapeutic effects.

Table 1 outlines the current innovative therapeutic approaches for aortic valve disease that are under clinical or preclinical investigation. Because most devices are still in their early stages of development, it remains challenging to determine which device will ultimately prove to be the most effective. However, a minimally invasive approach with demonstrably improved patient outcomes remains the primary focus. Among these, shockwave-based technologies, particularly NIUT, hold significant promise based on emerging data.

**Table 1:** Innovative Aortic Valve Therapeutic Approaches

Key Technology	Invasive				Noninvasive
	Lithotripsy	Histotripsy	Leaflet Scoring	Leaflet Splitting	NIUT
Mechanism	Unfocused shockwave emitted from a lithotripsy balloon. The pressure peak amplitudes on the edge of balloon to be 5∼20 Mpa	Ultrasonic shockwaves generated with piezoelectric generators. 2 frequencies: one about 30× greater than the other and avoid thermal injury	An expander that pushes the native aortic leaflets toward the frame for the creation of scoring lines. Designed to segment restrictive deposits and thereby increase leaflet mobility	Split/removes leaflets using specific devices eased by mechanical or electronic force. Mainly designed for coronary reaccess and prevention of coronary obstruction	Precisely focused pulse cavitational ultrasound delivered toward a predefined target within the aortic valve tissue. Pressure peak amplitudes at the focal spot to be up to 70 MPa
Status	Clinical study 10+ patients enrolled	Preclinical investigation	Clinical study 20+ patients enrolled	Clinical study 100+ patients enrolled	Clinical study 40+ patients enrolled
Follow-up	6 mo	—	1 y	1 y	6 mo
Retreatment	Yes	Yes	Yes	No	Yes
Current products and techniques	ShockWave (Shockwave Medical) TaurusWave (Peijia Medical)	Gemini (AorticLab)	Leaflex (Pi-Cardia)	ShortCut (Pi-Cardia) CATHEDRAL/BASILICA/UNICORN	Valvosoft (Cardiawave)

CATHEDRAL = CATHeter Electrosurgical Debulking and RemovAL; NIUT = noninvasive ultrasound therapy.

In conclusion, although the novel treatment approaches for calcific aortic stenosis, particularly those avoiding implantation or replacement, are still in their early stages, interventional cardiologists are seeing this hopeful light coming from the new horizon.

## Funding Support and Author Disclosures

Financial support was provided by Natural Science Foundation of Zhejiang Province (LQ22H020008), National Key R and D Program of China (2020YFC2008100), Healthy Zhejiang One Million People Cohort (K-20230085), and Zhejiang Province Science and Technology Department Key R and D Program (2022C03063). The authors have reported that they have no relationships relevant to the contents of this paper to disclose.

## References

[1] Xu H., Liu Q., Cao K. (2022). Distribution, characteristics, and management of older patients with valvular heart disease in China: China-DVD Study. JACC Asia.

[2] Timmis A., Vardas P., Townsend N. (2022). European Society of Cardiology: cardiovascular disease statistics 2021. Eur Heart J.

[3] Villemain O., Robin J., Bel A. (2017). Pulsed cavitational ultrasound softening: a new non-invasive therapeutic approach of calcified bioprosthetic valve stenosis. JACC Basic Transl Sci.

[4] Messas E., Ijsselmuiden A., Trifunovic-Zamaklar D. (2023). Treatment of severe symptomatic aortic valve stenosis using non-invasive ultrasound therapy: a cohort study. Lancet.

[5] Wang J.A., Liu X.B., Zhu Q.F. (2023). Novel transcatheter intervention on calcific aortic valve stenosis using shockwave technique: 2 case reports. Zhonghua xin xue guan bing za zhi.

[6] Bernava G., Fermi E., Gelpi G. (2022). Lithotripsy of calcified aortic valve leaflets by a novel ultrasound transcatheter-based device. Front Cardiovasc Med.

[7] Baumbach A., Mylotte D., Hildick-Smith D. (2021). Non-implant valve repair for calcific aortic stenosis: the Leaflex study. EuroIntervention.

[8] Babaliaros V.C., Gleason P.T., Xie J.X. (2022). Toward transcatheter leaflet removal with the CATHEDRAL procedure: CATHeter Electrosurgical Debulking and RemovAL. JACC Cardiovasc Interv.

[9] Tchétché D., Kodali S.K., Dvir D. (2022). First dedicated transcatheter leaflet splitting device: the ShortCut device. EuroIntervention.

[10] Mangieri A., Richter I., Gitto M. (2024). Chimney stenting vs BASILICA for prevention of acute coronary obstruction during transcatheter aortic valve replacement. JACC Cardiovasc Interv.

